# Unexplored dimension of shift work: the effects of late-early shifts on nurses and their wellbeing — a qualitative study

**DOI:** 10.1186/s12912-025-04173-8

**Published:** 2025-12-06

**Authors:** Uchita Karki, Hila Ariela Dafny

**Affiliations:** 1 College of Nursing and Health Sciences, Flinders University, Adelaide, South Australia Australia; 2 Caring Futures Institute, Flinders University, Sturt Road, Bedford Park, SA Australia

**Keywords:** Focus groups, Nursing staff, Hospital, Rotating shift work, Occupational stress, Job satisfaction, Anxiety, Sleep-quality, Wellbeing, Mental health

## Abstract

**Aim:**

To investigate nurses’ perceptions of the effects of late-early shifts on their overall wellbeing including physical and mental health.

**Background:**

Nurses work long hour shifts with short breaks in between to provide 24-hour care in a healthcare setting. Little is known about nurses’ perception of counterclockwise (CCW) rotating shifts, mainly the late-early shift. This study delves into nurses’ views on late-early shifts in three South Australian private hospitals.

**Design:**

An exploratory, qualitative design.

**Methods:**

Four focus group interviews, each with five nurses from three private hospitals in South Australia, were conducted post-ethical approval. Qualitative data underwent manual thematic analysis using an inductive approach.

**Results:**

Participants reported impacts on physical and mental health, unhealthy food habits, and social and work environment challenges associated with late-early shifts. These effects may be mitigated through interventions like extended breaks between shifts, reduced workload, and additional support.

**Conclusions:**

The late-early shift has detrimental effects on nurses’ wellbeing. This led nurses to opt for casual work, practice “quiet quitting,” or pursue nursing roles that accommodate their preferred shift schedules.

**Implications for nursing management:**

Research indicates that well-rested nurses provide quality patient care and improve health outcomes. Optimal scheduling and incentives play a vital role in enhancing nurses’ health and retention of the nursing workforce.

**No patient or public contribution:**

This paper explicitly explores the experience of nurses working late-early shifts in private hospitals in South Australia.

**Clinical trial number:**

Not applicable.

## Abstract (key message)

**What is already known about the topic?:**

•Shift work negatively affects nurses’ physical and mental health, leading to sleep disturbances, fatigue, and burnout.

•Fatigued nurses are more prone to errors and have diminished capacity to provide optimal patient care.

•Counterclockwise rotating shifts, such as the late-early shift, have been underexplored, particularly regarding their impact on nurses’ wellbeing.

**What this paper adds?:**

•This study offers qualitative insights into the perceived effects of late-early (Counterclockwise rotating) shift on nurses’ physical and mental wellbeing, job satisfaction, and personal lives.

•Highlights how late-early shifts affect nurses’ shift preferences and employment decisions, helping build an evidence base for healthcare organisations aiming to address these challenges.

•Suggests possible interventions, such as longer breaks and shift scheduling adjustments, to mitigate the negative impacts of late-early shifts on nurse wellbeing.

## Introduction

Shift work is outside the standard daytime work hours between 8 am and 6 pm, including rotating shift work, night shifts, and evening work [1]. Shift work is associated with adverse physical and psychological effects, as human physiology has biologically adapted to synchronise with the light–dark cycle [2]. A late-early shift is defined as an arrangement of shift work where nurses work a late shift followed by a morning shift the next day [3] commonly referred to as a quick return [4]. In South Australian private hospitals, late shifts start at (1415–1430) hours and end at (2215–2230) hours, whereas early shifts begin at (0645–0700) hours and end at (1445–1500) hours, allowing less than 9 h of break in between the late and early shifts. It is common for hospital nurses to work on rapidly rotating shift systems, which may vary by the direction of rotation. They may follow a clockwise (CW) (forwards) rotation direction (such as day, afternoon, night) or a counterclockwise (CCW) (backwards) rotation direction (such as day, night, afternoon). The rotational shift work impacts nurses’ physical and mental health and their wellbeing, as it leads to increased sleep disturbances, fatigue, emotional distress, reduced cognitive functioning and difficulties maintaining personal and social relationships [5].

World Health Organization (WHO) defines wellbeing as a mental, physical and social state, not simply the absence of infirmity or disease [6]. It is the ability to flourish, promoting a ‘good’ life where individuals are healthy, happy, capable and engaged [7]. Wellbeing is a combined definition of functioning well and feeling good [8]. It is conceptualised as a spectrum with high wellbeing, flourishing, and happiness at one end and low wellbeing, increased anxiety, and depression at the other end [9, 10]. High wellbeing portrays the experience of positive emotions and having a sense of purpose and control [8]. Conversely, poor mental health may be related to rapid social change, stressful work conditions, gender discrimination, social exclusion, unhealthy lifestyle, physical ill health, and human rights violations, where specific psychological and personality factors can make people vulnerable to mental health problems [11, 12].

In the workplace context, poor mental health includes stress, anxiety, depression, and syndromes such as burnout and compassion fatigue [13–15]. Burnout is a prolonged response caused by chronic exposure to interpersonal stressors within the workplace. Burnout is a psychological syndrome consisting of three dimensions: exhaustion, cynicism, and a sense of ineffectiveness [16]. Exhaustion was described as the loss of energy, wearing out, depletion, fatigue, and debilitation, whereas cynicism portrays irritability, withdrawal, inappropriate attitudes towards patients, and loss of idealism [16]. Burnout and work engagement negatively influence mental and physical health [17]. Other contextual challenges in the workplace, such as high workload, shift work, and insufficient support can act as barriers to nurses’ participation in interventions designed to improve mental health and wellbeing [18].

## Background

Nurses comprise Australia’s largest clinical workforce [19]. Long-hour shifts with insufficient breaks between them jeopardise nurses’ health by limiting adequate rest and sleep, ultimately compromising patient care and safety [20, 21]. Compromised sleep contributes to longer reaction times, reduced alertness, impaired judgment, decreased concentration, and forgetfulness, all of which increase the risk of medical errors among nurses [22]. Zhai et al. [23]. discovered a strong connection between poor sleep and an elevated risk of depression. Counter-clockwise (CCW) rotating or backward-rotating shifts deprive nurses of proper rest and eating nutritious foods, which in turn raises their stress and caffeine usage [20].

Hospitals and health care services adopt different shift patterns to run round-the-clock care. One of the various shift arrangements employed to cover the continuous demands of patient care includes CCW rotating shifts, particularly the late-early shift [3]. As recommended by Kennedy [3] and Safe Work Australia [24] optimal shift scheduling emphasises a minimum of 11 to 12 h between two shifts to safeguard against fatigue risks. It underscores the importance of breaks providing ample time for commuting, meals, and, crucially, rest, including sleep. However, in South Australian private hospitals, late shifts start at (1415–1430) hours and end at (2215–2230) hours, whereas early shifts begin at (0645–0700) hours and end at (1445–1500) hours, allowing only 8.5 h of break in between the late and early shifts. In line with recommendations [20], forward rotation from early to late and from late to the night shift is advocated to align with the body’s circadian rhythm. Proper work scheduling emerges as a pivotal factor in optimising nurses’ health, ensuring both their wellbeing and the quality of patient care [22].

Despite numerous existing literatures examining various aspects of shift work and nurses’ wellbeing [5, 21, 22, 25–27] there is a notable research gap concerning the effects of late-early shift patterns on nurses’ wellbeing, in particular, a lack of current evidence, especially within the South Australian private healthcare system. In addition, existing studies [26–28] predominantly focus on quantitative methods to examine the various aspects of shift work, while this utilises a qualitative method to examine the nurses’ perception on the effect of late-early shifts on their wellbeing physical and mental health by taking a comprehensive approach, considering the physical, mental, and social dimensions of wellbeing [29].

## The study

### Aim

The aim of this study was to investigate nurses’ perceptions of the effects of late-early shifts on their wellbeing. The insights gained from nurses’ perceptions can assist nursing leaders and policymakers in creating and implementing strategies to alleviate the challenges faced by nurses working late-early shifts.

### Research question

Among nurses working in private hospitals in South Australia, how does working late early shift influence their overall wellbeing?

## Methodology

### Design

An exploratory qualitative research design was conducted with four focus group interviews, with five participants in each group. Thematic analysis was used as a systematic and rigorous method to identify recurring patterns and underlying themes within the qualitative data [30].

### Study setting and recruitment

The study was conducted at the end of 2022 in three private hospitals in South Australia. Four focus group interviews were conducted with five participants in each group. Participants were recruited by advertising on two social media platforms (Facebook and LinkedIn). Nurses willing to participate contacted the researcher for participation in the respective social media platform. Participants received a written project overview and the researchers’ contact information before the focus group interview. Each focus-group interview was a mixed group of nurses from three different hospitals. The interviews were audio taped and then transcribed. Participants were offered a $30 gift voucher as a gesture of appreciation for their valuable contribution to the study.

Given that this study was conducted at the end of 2022, shortly after South Australia lifted its COVID-19 state of emergency in May 2022 [31], precautions remained in place, particularly within healthcare settings. However, most public restrictions had eased. Health-care workers continued to face exposure risks and were expected to adhere to workplace health directives, including staying home if unwell. To accommodate this and ensure participant safety, nurses were given the option to attend the focus group interviews via Zoom if they were experiencing any symptoms of illness. This flexibility aimed to support inclusivity while minimising any disruption to data collection due to lingering health concerns or workplace policies related to COVID-19. During the in-person interviews, participants were seated with adequate spacing, and hand sanitiser was made available. The allocation of participants into four focus groups was based on participant availability and scheduling preferences, rather than grouping by hospital or unit.

### Participant characteristics

A total of 20 registered and enrolled nurses working in medical wards, surgical wards and intensive care units participated in this study. The demographic details of this study’s participants are provided in Table 1. The participants’ ages ranged from 23 to 55 years. The sample consists entirely of female nurses with varying levels of professional experience, ranging from 1 to 25 years. Of the 20 participants, 14 worked full-time, while six were engaged as casual staff. Educational backgrounds revealed a diverse range, with two nurses holding diplomas in nursing, 14 possessing bachelor’s degrees, and 4 achieving the highest qualification of a master’s degree as demonstrated in Table 1.

**Table 1:** Demographic details of the participants

Participants	Age	RN/EN	Education	Experience In years	Private Hospital
FG1A	32	RN	MN	6	A
FG1B	32	RN	MN	4	B
FG1C	25	RN	BN	3	A
FG1D	32	RN	BN	7	C
FG1E	23	RN	BN	1	B
FG2A	55	RN	BN	25	C
FG2B	38	RN	BN	14	C
FG2C	30	RN	BN	10	B
FG2D	34	RN	MN	10	A
FG2E	33	RN	BN	9	C
FG3A	25	RN	BN	1	B
FG3B	29	RN	BN	6	B
FG3C	24	EN	DN	1.5	B
FG3D	23	RN	BN	1	A
FG3E	26	RN	BN	4	C
FG4A	53	EN	DN	16	C
FG4B	36	RN	MN	11	A
FG4C	26	RN	BN	4	B
FG4D	24	RN	BN	3	B
FG4E	27	RN	BN	5	A

RN = Registered Nurse; EN = Enrolled Nurse. MN = Master of Nursing; BN = Bachelor of Nursing; DN = Diploma of Nursing

### Inclusion and exclusion criteria

The inclusion criteria for this research included registered and enrolled nurses who expressed interest in participating and willingly provided consent. Additionally, eligible participants were required to be actively employed in a private hospital in South Australia, having completed at least one late-early shift within the past month. While the study was advertised on social media, only nurses from private hospitals were included due to the unique scheduling of late-early shifts in these settings, where there is only an 8.5-hour break between shifts, compared to the longer breaks in public hospitals. Exclusion criteria included individuals unwilling to participate in the study, as well as nurses who had not worked a late-early shift or had less than a month of experience as a nurse. Lastly, nurses working in specialised areas like day surgery units, where late-early shifts were not part of their regular duties, were also excluded from participation. These criteria aimed to ensure a targeted and relevant participant group for investigating the effects of late-early shifts on nurses’ well-being in South Australian private hospitals.

### Data collection

All focus group interviews were conducted face-to-face. Before beginning, participants completed a demographic information form and were reminded that their participation was voluntary, with the option to withdraw at any time without consequences.

To protect confidentiality and encourage open dialogue, participants were reminded at the start of each focus group to respect each other’s privacy and avoid sharing identifiable information outside the group. Consent forms outlined confidentiality expectations, and ground rules were established to promote a respectful, non-judgmental environment. Participants were encouraged to speak in general terms, and identifying details were removed during transcription. Importantly, no focus group included both managerial-level staff and junior nurses, or individuals in a direct reporting relationship, to minimise power imbalances and reduce the risk of professional repercussions. Group allocation was based on availability, ensuring a diverse yet non-hierarchical mix of nurses from different units and hospitals.

A semi-structured interview guide, developed through a review of the literature and aligned with the study’s research questions, was used to facilitate the discussions. While core questions provided structure, the format remained flexible, allowing new themes and issues to emerge naturally during the conversations.

Open-ended questions were used to explore nurses’ perceptions of working late-early shifts.

- How would you describe your experience working late-early shifts?
- What do you think are the benefits of Late-early shift, if any?
- What do you think are the advantages/disadvantages of Late-early shifts, if any?
- According to your experience, how is the late-early shift different from any other pattern of shift work like early-late, late-late or early-early?
- If you were allowed to choose all your shifts, how often would you work late-early shift and why?
- Are there any factors that make late-early shifts better or worse? What are they?
- How does working a late-early shift impact your personal life?
- How does working a late-early shift impact your profession as a nurse?
- How does working a late-early shift impact your family and friends?
- Do you have any suggestions regarding working late-early shifts?

Every focus group interview commenced with these questions, after which additional topics were delved into based on issues brought up by the nurse. The four focus group sessions ranged from 45 min to 60 min and were held outside the hospital setting to provide a comfortable environment for the participants. The semi-structured focus groups were held between August and September 2022. Each focus group interview was recorded using two audio devices to ensure all participants’ contributions were clearly captured. Consent to record the sessions was obtained from participants and interviews were transcribed verbatim.

### Data analysis

Obtained data was thematically analysed manually using an inductive approach based on a six-phase guide by Braun & Clark [30], that of six steps: familiarisation with data, generating initial codes, searching for themes, reviewing themes, defining and naming themes and producing the report. A total of four themes and eleven subthemes were identified. The first author carried out the initial coding of several responses into themes. Following a thorough review of the literature and collaborative discussions, both authors identified and integrated additional themes, finalising the thematic framework by mutual agreement.

### Rigour and reflexivity

Rigour was ensured through a systematic approach, employing purposive sampling to target registered and enrolled nurses with recent experience in late-early shifts. This strategic sampling aimed to provide relevant and in-depth data for a nuanced exploration of the research questions. Overall rigour was enhanced by having both researchers independently examine the data. The authors held collaborative discussions to review the emerging data, which led to consensus on the key themes, thereby contributing to the credibility and dependability of the findings in line with Guba and Lincoln’s [32] for trustworthiness in qualitative research.

Regular reflections and team discussions facilitated identifying and bracketing preconceptions or assumptions that could impact the interpretation of results. This practice aligns with a Husserlian phenomenological approach, which emphasises the suspension of researcher biases to attend to participants’ lived experiences as they are [33] Transparency in reporting was integral to reflexivity, explicitly detailing inclusion criteria, participant recruitment, and data analysis procedures in the research documentation. By providing a clear account of methodological choices and acknowledging potential subjectivities, the researchers aimed to enhance the study’s trustworthiness and transferability [32].

## Findings

**Fig. 1:**
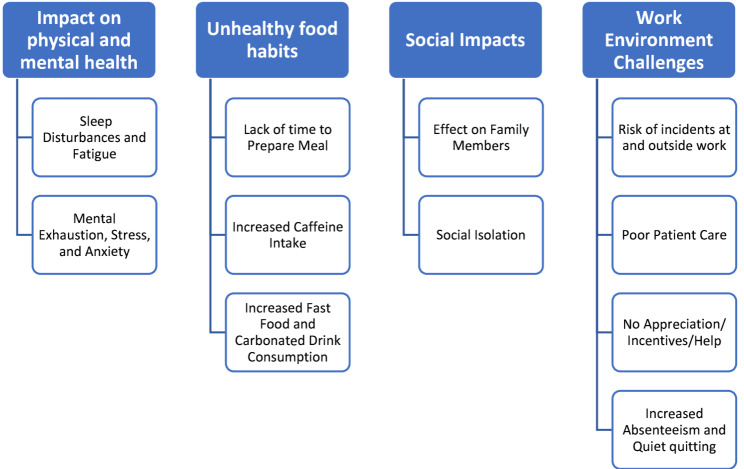
Themes and Subthemes identified

Nurses described their experience of working late-early shifts as physically tiring and mentally exhausting, causing negative impacts on their family, social life, safety at work, and wellbeing. Four themes were identified from the four focus groups, as indicated in Fig. 1 (Themes and Subthemes identified), and include the following: (1) Impact on physical and mental health, (2) Unhealthy food habits, (3) Social impacts and (4) Work environment challenges.

### Impact on physical and mental health

Nurses consistently highlighted the late-early shift’s impact on their physical and mental health. Sleep disturbances, fatigue, mental exhaustion, and heightened stress emerged as predominant concerns, shedding light on the profound challenges of late-early shifts.

#### Sleep disturbances and fatigue

Nurses reported sleep disturbances as a prominent impact of the late-early shift, with unanimous agreement on the struggle to sleep between the late and early shifts. Some nurses experienced prolonged sleep disturbances lasting more than a week after a single late-early shift, contributing to apprehension about having to work multiple such shifts.

*I think I have got insomnia now. After late-early shift although I go to bed as early as possible I hardy fall asleep by 1am. 4–5 hours of sleep is not enough to go back to the same stressful job again in the morning. -FG2B*

*There have been days when I had to go to work without getting any sleep. -FG1B*

*For me*,* only one late early a fortnight can affect my whole fortnight sleep pattern. -FG1D*

The mandated 8–8.5 h break between late and early shifts did not account for travel time and the necessary downtime for the mind to settle before sleep. The absence of sufficient rest increased tiredness, making it challenging for nurses to exercise regularly or maintain physical fitness.

*We work two shifts in short amount of time which is being on our feet for a long time with short breaks in between. I get sore feet and back*,* after a late early shift I always get this headache. I call it a late-early headache! - FG3A*

*I fall asleep on the couch sometimes after late-earlys because I am too exhausted*,* I can’t make dinner or go on a walk or go to the gym to keep myself fit. - FG4D*

#### Mental Exhaustion, Stress, and anxiety

Nurses reported experiencing mental exhaustion, stress, and anxiety due to various factors, including sleep disturbances, insufficient time for personal and family matters, and the challenge of working diligently in stressful situations despite being already tired. Participants described vivid dreams related to their work, highlighting the mental toll of continuous pressure with minimal rest.

*Sometimes when I finally fall asleep*,* I dream of patients ringing the bell and myself rushing to get things done. It is very stressful. - FG3B*

*On the morning shift after late shift I always find that at 1130–1200 I am mentally suffering…. You know…I can’t make simple decisions and I forget things* a lot! …*and by that time if I waited another hour for lunch! It only gets worse!*- *FG2A*

Stressful morning shifts after the afternoon shift, difficulty making decisions, and heightened forgetfulness were reported, emphasising the strain on mental well-being. Despite facing physical and mental fatigue, nurses felt compelled to continue working, expressing a sense of obligation despite the toll on their overall health.

*Our mental health is at risk due to the continuous pressure with minimal rest in between - FG1D*

*I describe my night in between late and early shift as*,* what’s the word?…. as a night full of anxiety like anxiety of missing 3 alarms you have set up. - FG4C*

*You feel like you can’t give your 100% mentally and physically. But you got to do what you got to do! FG2B*

In addition to physical and mental fatigue, unhealthy food habits, such as an increase in the consumption of caffeine, carbonated beverages, along with fast food, were reported to be linked with late-early shifts.

### Unhealthy food habits

Nurses identified a lack of time for meal preparation as a primary factor contributing to unhealthy food habits. The demands of their schedules led to an increased reliance on quick and convenient options, resulting in higher consumption of caffeine, carbonated drinks, and fast food. The time constraints placed on nurses affected not only the nutritional quality of their meals but also introduced habits that could impact their overall health and well-being.

### Lack of time to prepare meals

The identified link between insufficient time and poor dietary choices highlights nurses’ broader challenges in maintaining a healthy nutritional balance amid demanding work schedules.

*I live alone*,* and I get very little time to prepare my meals between late-early shifts. I think that could lead to poor nutrition among nurses. FG1C*

*I lose my appetite when I work the late-early shift. Sometimes I don’t eat the whole day and regret it later. It’s like you can either rest or eat. I’m so exhausted I crash on my couch and fall asleep! FG4B*

Some nurses reported losing their appetite during late-early shifts, leading to regrettable patterns of not eating throughout the day due to exhaustion. In instances of consecutive late-early shifts, nurses acknowledged a reliance on fast food for the entire week.

#### Increased caffeine, carbonated beverages and fast-food intake

In addition to consuming fast food due to insufficient meal preparation time, nurses experienced increased caffeine and carbonated beverages intake during late-early shifts. Participants acknowledged increased caffeine consumption during late-early shifts, with individuals reporting an elevated coffee and diet coke intake to stay awake and combat fatigue.

*If you work 2 late-earlys in a row*,* that is your week of fast food. FG3D*

*My intake of coffee and diet coke goes up! I need something to keep myself awake! FG2A*

*You drink 2–3 cups of coffee throughout your shift. Your heart is racing*,* and you are not thinking straight*,* and you have a patient to look after*,* is that even*,* right? FG2E*

### Social impact

Nurses reported declining quality of their social life and a visible effect on them and their family members as a result of working late-early shifts.

#### Effect on family members

The late-early shift significantly affected nurses’ family lives, preventing them from spending quality time with their spouses and children. Nurses reported often missing seeing their family members for extended periods, with some experiencing a 24–36-hour gap due to overlapping work and sleep schedules. Participants experienced that their spouses also had to deal with sleep disturbances as they woke them up while getting ready to go to work in the morning and returning from work. Nurses also expressed guilt, particularly regarding breastfeeding babies, as the demands of the late-early shift hindered their ability to provide sufficient time and attention to their families.

*I have a breast-feeding baby. She wakes up at night 2–3 times. I am guilty that I can’t give her enough time*,* but I am so tired that sometimes I get so irritated when she wakes me up in between my late-early shift. FG2C*

*When I am struggling to sleep and fidgeting in the bed*,* I wake my husband up sometimes. His sleep routine gets affected too. Having multiple alarms set up and getting up early in the morning to rush to work*,* I think your spouse suffers with you! FG1B*

*My children are at home when I work late-early*,* I sometimes don’t see them for 36 hours due to school and work. It is you know… quite heart-breaking for a mum and not a pleasant experience for my children too. FG2A*

#### Social isolation

The late-early shift in nursing introduced physical exhaustion and became a reason for social isolation among nurses. Despite having time after late-early shifts, nurses often felt too tired, impatient, and annoyed to engage in social activities. The necessity to recharge for the next day’s responsibilities further emphasised the choice to prioritise rest over social activities.

*Nursing is a very stressful job. I must talk to my friends and family back home to vent. Late early takes away my chance to talk to them and if you do few in a row*,* you just work*,* come home*,* try to sleep and go back to work the next day again. It gets lonely at times. FG1C*

*My patience level is really low when I am exhausted*,* I could think this patient is such a pain! Same with husband*,* kids*,* buddy nurses and whoever! I end up choosing to nap rather than going out. FG4B*:

*If I was working late early*,* I would not have been able to attend this interview. Same with other social stuff*,* rest becomes the priority. FG3E*

### Work environment challenges

Nurses unanimously recognised the dangerous consequences of late-early shifts on their ability to function safely, within and outside the workplace. Increased stress and vigilance were associated with apprehension of losing their professional registration. Diminished Job satisfaction was noted as nurses struggled to provide optimal patient care. Additionally, it was reported that the lack of recognition, incentives, and support further exacerbated dissatisfaction, contributing to a phenomenon termed “quiet quitting,” wherein nurses disengage and consider leaving their roles due to the taxing nature of late-early shifts.

#### Risk of incidents at and outside work

Nurses expressed concerns about compromised concentration and increased risk of errors. Participants noted that quick return to the early shift after the late shift posed challenges to maintaining focus, increasing the need for meticulous cross-checking, especially in medication administration. The fear of making mistakes and its potential impact on professional registration emerged as a significant source of stress. Furthermore, participants highlighted the risk of incidents during commuting. Driving to an early shift after insufficient sleep overnight was particularly challenging, with nurses expressing fear of falling asleep at the wheel. The apprehension of adverse consequences in terms of patient care and personal safety underscored the pervasive impact of late-early shifts on nurses’ well-being and professional performance.

*I find it dangerous driving to work*,* and it is a big one you know! I put down my window*,* have music on and sit upright. FG2C*:

*Sometimes*,* when I have not slept well*,* I ask my husband to drop me off at work because I don’t trust myself falling asleep while driving. However*,* my husband can’t work on my behalf. I am not 100% sure no incidents at the workplace will be caused. FG4D*

*One of the cons of late-early shift is that you have a hard time focusing. There is an increased risk of errors*,* like medication errors! You must stay completely alert to avoid mistakes. I work hard and further exhaust my already tired brain because I am scared of losing my registration. FG1E*:

*I must confess that the late shift is not a problem when yo work late early. The early shift is tricky. You are checking a medication 5 times just because you don’t trust your brain that hasn’t had enough rest. FG3C*

#### Poor patient care

The study revealed a consistent and compelling theme among nurses regarding decreased job satisfaction associated with difficulties in providing optimum care. Participants reported that their ability to deliver quality patient care significantly diminished due to fatigued cognitive abilities, physical exhaustion, irritability and inability to participate in effective communication. Nurses conveyed that their ability to deliver superior patient care improved when they were adequately rested and could attend to their own wellbeing.

While the continuity of patient allocation between shifts emerged as a potential advantage, this was not always guaranteed.

*When you do it for the long term*,* it is not healthy for your body and not healthy for your profession as it takes away the ability to provide quality nursing care. FG2C*

*Getting the same patients to look after helps*,* but that hasn’t always been the case for me. When they are not allocating the same patients to us*,* they are taking away our ability to provide better care. FG1A*

*Having the same patient is the only help we get if we are lucky*,* it does help to an extent. I know my patients*,* and they know me. However*,* they don’t know I am low on my tolerance and crankier in the morning. Sometimes patients feel pity for us that we have to come back in such a short amount of time. FG2B*

*One can tell by looking at their faces that they are back in the morning after a late shift. They look tired*,* they are yawning most of the time*,* they talk less*,* are not smiling and can get angry for no reason. At the end of the day*,* it affects teamwork and patient care. FG1B*

#### No appreciation, incentives and support

Nurses expressed a decline in job satisfaction due to challenges in providing quality care and a lack of motivation stemming from insufficient appreciation, recognition, incentives, and support. Participants in the focus groups acknowledged the difficulty of completing two shifts in a short span. Also, they expressed frustration over the toll it takes on their physical and mental wellbeing, compounded by a perceived absence of appreciation. The desire for alternatives to late-early shifts was evident, with suggestions for incentives, longer breaks, and additional assistance to mitigate the associated challenges. Overall, the dissatisfaction among nurses was linked to the nature of the shifts and a perceived lack of acknowledgment and tangible support from the organisational management.

*I have a love-hate relationship with late early shifts. I love how I finish two shifts in a short amount of time. I hate how I do that at the cost of my body and mind with no appreciation. FG2D*

*I would never choose to do a late early shift If I was given a chance. It would have been different if there was a provision of incentives*,* longer break in between and extra hand to help. FG4E*

*It would be helpful if we were provided nutritious food as an incentive for having no time to prepare it. This should be the least the management can do for our wellbeing. FG1C*

#### Quiet quitting

Nurses expressed a lack of motivation to exceed minimum job requirements, opting for the bare minimum and actively seeking employment opportunities where their shift preferences are considered. This trend aligned with the concept of “quiet quitting,” where nurses, feeling fatigued and underappreciated, opt for the bare minimum and exit the workplace. This phenomenon was highlighted in discussions, emphasising how the cycle of nurses leaving harms the nursing team’s skill mix. The resulting inadequate skill mix on the floor hampers the work of those nurses who choose to stay, contributing to a less-than-ideal team dynamic. The frustration with this situation was evident, as nurses expressed concern over the high turnover and shortages of staff, attributing it to factors such as the imposition of late-early shifts and a lack of consideration for individual preferences.

*You will probably take sick leave more than you would if you work late early. FG1E*

*The other day*,* I heard this term called quiet quitting in TikTok. I think it is common among nurses too. They just do the bare minimum and go home. No one is going out of their way to contribute significantly. I guess it is so because they are tired and not appreciated. FG1A*

*A good skill mix is necessary to build up a good team. Nurses are quitting*,* choosing to go casual*,* or choosing areas where they don’t have to do shift work. Sometimes*,* we can see all the junior nurses working in a shift. It is not an ideal team.; FG3D*

*I can’t do this long term. I might change the job or even the field. It is not necessary for nurses to do late-early shifts. At private hospitals*,* the staffing is tight. High nurse turnover and shortage could be somewhat addressed by giving nurses a shift of their preference. FG1B*

## Discussion

The finding of this study indicates that nurses working consecutive late-early shift rotations, commonly referred to as “quick returns” with less than 11 h of rest, experience significantly poorer wellbeing outcomes. These compressed rosters were associated with increased fatigue, impaired sleep, and elevated stress levels. Such findings are consistent with previous research linking quick-return schedules to negative outcomes. For example [34], found that newly graduated nurses experienced higher stress during weeks involving more quick returns. Similarly, It was reported that frequent quick returns significantly reduced sleep duration and, when coupled with lower work motivation, contributed to burnout [4]. In addition, commuting after insufficient sleep was perceived as physically taxing and unsafe, reflecting findings that excessive sleepiness among shift workers increases the risk of road and occupational accidents [25].

These findings align with the broader literature on shift work and nurse health. Rotating and irregular shifts have been consistently shown to compromise wellbeing. For instance, a multi-site study conducted in Spain reported that nurses working rotating shifts exhibited lower sleep efficiency and higher psychological distress such as depersonalisation, anxiety, and gastrointestinal symptoms [35]. Likewise, a study by Han et al. [36]. highlighted that nurses operating under current shift systems frequently face intense conditions and profound physical and mental fatigue. Importantly, the review concluded that many existing rostering practices are structured in ways that are detrimental to nurses’ health, performance, and patient care. The present study reaffirms and sharpens these concerns by demonstrating that specific scheduling practices like late-early shifts are correlated with measurable declines in not only nurses’ wellbeing but also patient’s safety. This suggests that the adverse effects of shift work stem not only from night shifts or extended hours, but also from the timing and insufficient recovery periods between consecutive shifts.

Unlike previous studies that often compare fixed and rotating shifts or 8-hour and 12-hour rosters, this investigation emphasises the importance of intra-shift turnaround times. These results complement the findings of a large randomised controlled trial [37], that demonstrated how halving the frequency of quick returns modestly improved insomnia and daytime sleepiness. Collectively, this growing body of evidence underscores the tangible benefits of limiting quick returns. In the current study, some participants reported feeling nearly “sleep deprived” despite working standard-length shift highlighting the disproportionate impact of compressed recovery time.

Importantly, the discussion foregrounds the practical implications of these findings. The consistency of the results with established risks associated with shift work suggests several actionable strategies. Foremost among these is the need to ensure sufficient rest between shifts. This approach is supported both by regulations such as the European Union’s mandate for a minimum of 11 h rest between shifts and by empirical evidence linking quick returns to accidents, absenteeism, and fatigue [34]. The findings further support recommendations that organisations adopt rostering structures that minimise quick returns and promote longer sleep durations among nursing staff [4]. Embedding such scheduling practices could directly mitigate the fatigue, insomnia, and operational disruptions observed in the current study.

Additionally, the findings highlight the importance of nurse involvement in roster planning. Evidence suggests that when staff have input into or control over their rosters (e.g., through self-scheduling), adaptation improves [38]. Empowering nurses with greater flexibility and autonomy in scheduling may help to offset the adverse impacts of unavoidable shifts. Finally, the high prevalence of fatigue and burnout reported underscores the need for broader systemic support. Organisational interventions such as resilience training programs, peer support mechanisms, and workload redistribution have shown promise in enhancing nurses’ wellbeing [39].

### Implications for policy and practice

The findings of this study highlight several actionable strategies to support nurses’ wellbeing.

### Optimise shift scheduling

Move to forward-rotating (clockwise) shift cycles and eliminate “quick returns.” For example, ensure at least an 11-hour break between a late shift and the following morning shift to allow physiological recovery and support sleep hygiene.

### Nutrition support

Ensure access to healthy food options during shifts. Shift work can disrupt regular eating patterns and lead to unhealthy dietary habits, often due to limited time for meal preparation between quick returns.

### Foster a supportive workplace culture

Healthcare institutions should recognise the challenges of late-early shifts and measures such as reduced workloads and introducing penalty rates for quick returns should be considered by health care institutions to optimise nurses’ wellbeing. Developing policies that acknowledge and accommodate nurses’ shift preferences may contribute to increased job satisfaction, potentially reducing the “quiet quitting” phenomenon and encouraging a more stable nursing workforce.

### Study strengths and limitations

The research focused on a nursing shiftwork pattern that had not been extensively studied. During interviews, nurses expressed a keen interest in sharing their experiences, demonstrating a desire to have their perspectives acknowledged and heard.

One of the limitations of this research is the absence of representation of male nurses. All the participants in this study were female. The absence of male participants may potentially influence the generalizability of the findings. Nonetheless, it is noteworthy that the gender composition of the Registered Nurse workforce, as reported by the Australian Government Department of Health [40], indicates a predominant female presence, with females constituting 88.6% of the workforce. Future studies, nevertheless, are encouraged to actively seek a more balanced representation to enhance the comprehensiveness and applicability of study outcomes.

Also, a more extensive study could be conducted to include nurses working in public hospitals and nursing homes to generalise some of the findings and find differences in the perceptions of nurses working in private hospitals and other healthcare settings. However, it was found that nurses in public hospitals get longer breaks between late-early shifts in comparison to nurses in private hospitals, which justifies the inclusion of nurses from private hospitals only in this study.

## Conclusion

This study explored nurses’ perceptions of the impact of the late-early shift on their wellbeing. The findings indicated that this scheduling pattern disrupts sleep and recovery time, resulting in chronic fatigue, heightened stress, and difficulty maintaining a balance between work and personal life. Nurses described disturbances to their circadian rhythms, limited opportunities for rest, and challenges in sustaining healthy routines. These impacts contribute to burnout and may compromise both patient safety and the quality of care.

Nurses are predisposed to adopting unhealthy eating habits due to exhaustion and insufficient time for meal preparation. While carbonated beverages and caffeine intake were increased to keep nurses awake. Also, nurses found it challenging to create a balance between their social life and work. Nurses indicated that the optimum scheduling focusing on the clockwise rotation of shifts could be helpful, along with longer breaks in between shifts that consider the travel time and time to prepare meals.

To address these issues, the study proposes several practical strategies. In addition to recognition, tangible incentives are vital for promoting nurses’ health and job satisfaction. Nurses highlighted the value of access to nutritious meals, adequate staffing support, and longer breaks between shifts to support physical and mental wellbeing. They also advocated for penalty rates for late-early shifts as both acknowledgment of their commitment and a financial incentive for maintaining quality care under demanding conditions.

### Clinical message

- This study contributes to the global literature exploring nurses’ perceptions of the effects of late-early shifts on nurses’ wellbeing.
- This study identifies areas that need improvement to ensure that safety at work is maintained and adequate support is available for nurses, which might subsequently contribute to the retention of nursing staff, job satisfaction and quality patient care.
- In response to the significant challenges encountered by nurses working late-early shifts, it is imperative for nursing leaders and policymakers to collaboratively devise and implement targeted strategies aimed at alleviating these difficulties.

## Data Availability

Data available on request from the authors - The data that support the findings of this study are available from the corresponding author, upon reasonable request.

## Abbreviations

WHO: World Health Organization
HREC: Human Research Ethics Committee
FG: Focus Group
RN: Registered Nurse
CCW: Counterclockwise
CW: Clockwise
